# Regulatory mechanism of pyrrolidine dithiocarbamate is mediated by nuclear factor-κB and inhibits neutrophil accumulation in ARDS mice

**DOI:** 10.3892/etm.2014.1738

**Published:** 2014-05-28

**Authors:** HONGMAN WANG, LISHENG XU, JIPING ZHAO, DONGHUI WANG, RANRAN GUO, JUNFEI WANG, WENBIN GONG, TIAN LIU, YUANYUAN ZHANG, LIANG DONG

**Affiliations:** 1 Department of Pulmonary Medicine, Qilu Hospital, Shandong University, Jinan, Shangdong 250012, P.R. China; 2 Department of Pulmonary Medicine, The Thrid Affiliated Hospital of Liaoning Medical University, Jinzhou, Liaoning 121001, P.R. China; 3 Department of Pulmonary Medicine, The Fifth Affiliated Hospital of Zunyi Medical University Zhuhai, Zhuhai, Guangdong 519100, P.R. China

**Keywords:** acute respiratory distress syndrome, CD11b/CD18, intercellular adhesion molecule-1, nuclear factor-κB, pyrrolidine dithiocarbamate

## Abstract

The aim of the present study was to investigate the regulatory mechanism of nuclear factor (NF)-κB on polymorphonuclear neutrophil (PMN) accumulation and the inflammatory response in lung tissues with acute respiratory distress syndrome (ARDS), as well as the therapeutic effect of pyrrolidine dithiocarbamate (PDTC). Mouse models of ARDS were established by intraperitoneal injection of lipopolysaccharide (LPS). BALB/c mice were divided into control, LPS and PDTC + LPS groups. The expression of PMN adhesion molecules, CD11b/CD18 and intercellular adhesion molecule-1 (ICAM-1), were detected by immunohistochemistry, while the protein expression levels of NF-κB p65 in the lung tissue were analyzed by western blot analysis. In addition, flow cytometry was used to investigate the apoptosis rate of PMNs in the bronchoalveolar fluid, and the expression levels of interleukin (IL)-1β, IL-8 and tumor necrosis factor (TNF)-α and myeloperoxidase (MPO) activity were also determined. Following an intraperitoneal injection of LPS, alveolar septum rupture, pulmonary interstitial hyperemia and PMN infiltration in the alveolar was observed. The protein expression of p65 in the pulmonary cytoplasm decreased, while the expression of p65 in the nucleus increased. The levels of IL-8, IL-1β and TNF-α increased and the high expression status was maintained for 24 h. As the time increased, CD11b/CD18 and ICAM-1 expression increased, as well as MPO activity, while the apoptosis of PMNs was delayed. Compared with the LPS group, the expression of p65 in the pulmonary cytoplasm and the PMN apoptosis rate increased following PDTC intervention, while the expression of p65 in the nucleus decreased, as well as the expression levels of the cytokines and MPO activity. Therefore, PDTC reduced the production of inflammatory cytokines via the NF-κB pathway, which reduced the activation of PMNs in the lung tissue and promoted PMN apoptosis.

## Introduction

The main pathological changes in acute respiratory distress syndrome (ARDS) are diffuse pulmonary interstitial and alveolar edema, caused by increased pulmonary microvascular permeability. The clinical fatality rate of ARDS is as high as 40–70% (1). The underlying pathogenesis of ARDS is excessively uncontrolled inflammatory responses that are mediated by various inflammatory cytokines, however, treatment has limited efficacy (2). Lipopolysaccharide (LPS) is the major component of the outer membrane of gram-negative bacteria and is a common trigger of sepsis, which is an important initiating factor in the activation of nuclear factor (NF)-κB (3). Previous studies have demonstrated that a large number of polymorphonuclear neutrophils (PMNs) accumulate in the lung tissue and release inflammatory cytokines, including interleukin (IL)-1β, IL-8 and tumor necrosis factor (TNF)-α, when the body fights against infection or trauma, which plays an important role in initiating and maintaining the inflammatory response (4,5). Research has often focused on the activation of monocyte-macrophage systems, which has gradually gained interest in the study of the pathogenesis of ARDS. PMNs are important effector cells in ARDS, and the infiltration and activation of PMNs involve a complex process that includes the recruitment, adhesion and chemotaxis of PMNs. In addition, the process is associated with PMN adhesion molecules CD11b/CD18, pulmonary vascular endothelial intercellular adhesion molecule-1 (ICAM-1) and PMN chemokines, such as IL-8. The expression of CD11b/CD18 plays an important role in PMN aggregation and activation (6). CD11b/CD18 is a heterodimer of the αM (CD11b) and β2 (CD18) subunits, which are key factors in the inflammatory response, as they combine with ICAM-1 and mediate adhesion between PMNs and microvascular endothelial cells, which then transmits an intracellular signal (7). CD11b/CD18 may be used as indicators of cell adhesion function (6). NF-κB is a critical nuclear transcription factor that activates the transcription of a number of inflammatory cytokine genes. The activation of NF-κB is closely associated with the overexpression of adhesion molecules, chemokines and other cytokines. The aggregation and migration of PMNs, which participate in the regulation of inflammation, plays an important role in ARDS (8).

Pyrrolidine dithiocarbamate (PDTC), an inhibitor of NF-κB, inhibits the expression of inflammatory cytokines, such as TNF-α and ILs, at the transcriptional level (9). In addition, PDTC reduces the expression of adhesion molecules on the surface of PMNs and endothelial cells and the accumulation of inflammatory cells in inflammatory sites (10,11). This reduces the release of myeloperoxidase (MPO) in the inflammatory sites, thus, decreases the damage to the body (12). Apoptosis is the main pathway of PMN clearance in the inflammatory response. PDTC may promote the apoptosis of PMN by inhibiting the activation of NF-κB (13).

Therefore, regulating the activation of NF-κB and inhibiting p65 subunit transfer to the nucleus may prevent the aggregation of PMNs and reduce the incidence of ARDS. The aim of the present study was to evaluate the direct effects of PDTC on PMN activity, characterized by the protein expression changes of NF-κB p65, the infiltration of PMNs and the excessive release of inflammatory cytokines.

## Materials and methods

### Animals

BALB/c mice (age, 6–8 weeks; weight, 20±2 g) were purchased from the Experimental Animal Center of Shandong University (Jinan, China). The experimental procedures were approved by the ethics review committee for animal studies at Qilu Hospital, Shandong University and according to animal welfare and all the animals experimental guidelines were followed. Mice were maintained at room temperature (24°C) with a 12:12 h light-dark cycle and allowed free access to water and standard laboratory chow.

### Survival ratio of the mice and experimental grouping

To assess the survival ratio, the mice received an intraperitoneal injection of 20 mg/kg LPS (*Escherichia coli* O55:B5; Sigma-Aldrich, St. Louis, MO, USA) with or without various intraperitoneal doses of PDTC (40, 120 or 160 mg/kg; L04358; ALIKESI, USA). PDTC was administered 30 min prior to the LPS challenge. The survival rates of the mice were recorded every 12 h for three days following LPS administration in each group (n=10).

To further study the protective effect of PDTC on mice treated with LPS, the mice were randomly divided into three groups: Control (20 ml/kg saline, i.p.), LPS (20 mg/kg LPS, i.p.) and PDTC + LPS (120 mg/kg PDTC, i.p. and 20 mg/kg LPS, i.p.). The mice were anesthetized by intraperitoneal injection of 10% chloral hydrate (3.5 ml/kg) and sacrificed using arotic phlebotomy at 4, 12 and 24 h.

### Histopathological analysis

The left lung was fixed with 4% paraformaldehyde for 24 h, embedded in paraffin and cut into 4-μm sections. Following staining with hematoxylin and eosin (Sigma-Aldrich), microscopic evaluation was performed to characterize the injury status. Lung injury was scored according to congestion, edema, interstitial inflammation and the aggregation of inflammatory cells. Each pathological feature was scored on a scale from 0 (normal) to 4 (severe). The total score was calculated by adding up the individual scores of each region.

### Lung wet to dry weight (W/D) ratio measurement

The right lung was removed and the wet weight was measured. Lung tissue was placed in an oven at 80°C for 72 h to obtain the dry weight. The W/D ratios of the lungs were calculated to quantify the degree of pulmonary edema.

### Lung permeability index (LPI)

The LPI was equal to the total protein in the bronchoalveolar fluid (BALF) divided by the total protein in the serum. The protein concentration in the BALF and serum was measured using the Bradford Assay (Thermo Fisher Scientific, Waltham, MA, USA) (14).

### Preparation of the BALF

BALF was collected and washed three times with 1.5 ml ice-cold phosphate-buffered saline (PBS) in all the groups. In each mouse, 80% (1.2 ml) of the total volume was recovered (15). Following centrifugation of the BALF at 1,700 × g for 7 min at 4°C, the supernatant was stored at −80°C for subsequent experiments.

The cell pellet in the BALF was resuspended in 1 ml red blood cell lysis buffer to eliminate the red cells. White cells in the BALF were then repelleted by centrifugation at 500 × g for 20 min at 4°C. The cell pellet was again suspended in PBS and the proportion of PMNs and living cells was adjusted to >95% by trypan blue staining.

### Extraction of protein in the cytoplasm and nucleus (16)

Lung tissue samples, weighing ~100 mg, were thoroughly washed with 0.01 M PBS, adding 1.5 ml nuclear protein extract lysis buffer A (BioTeke Corporation, Beijing, China). The samples were then placed on ice for 15–30 min and homogenized with an electric homogenizer following the addition of 0.5 ml ice cold 10% NP-40. Next, the samples were vortexed for 10 sec and centrifuged at 4°C and 12,000 ×g for 30 sec; the supernatant produced was the cytoplasm protein extract. The precipitate was washed once with cold PBS and centrifuged at 4°C and 12,000 × g for 30 sec, after which the supernatant was discarded. Next, 1.5 ml join nucleoprotein extract lysis buffer B (BioTeke Corporation) was added and the samples were placed on ice for 30 min. The samples were centrifuged again at 4°C and 12,000 g/min for 2 min to produce the aspirate supernatant (nucleoprotein). Using Bradford colorimetric determination, the nuclear protein concentration was adjusted to 0.5–1.0 μg/μl and the samples were placed and stored at −70°C.

### Measurement of NF-κB p65 protein expression by western blot analysis

Total protein was quantitated using the Pierce BCA Protein Assay kit (Thermo Fisher Scientific). Equal quantities of total protein (20 μg) were separated on 10% Bis-Tris gels in MOPS SDS Running Buffer (Invitrogen Life Technologies, Carlsbad, CA, USA) and transferred to polyvinylidene difluoride (PVDF) membranes (Immobilon; Millipore Corporation, Bedford, MA, USA). The PVDF membranes were blocked with 5% skim milk in Tris-buffered saline [TBS; 50 mM Tris-Cl (pH 7.5), 150 mM NaCl] for 60 min at room temperature and then incubated overnight with anti-pNF-κB p65 (1:200; Santa Cruz Biotechnology, Inc., Santa Cruz, CA, USA) or anti-β-actin antibodies (1:500; Santa Cruz Biotechnology, Inc.) in TBS-Tween-20 (TBST; 0.1% Tween-20). The membranes were washed three times with 1X TBST for 5 min, and then incubated for 1 h with secondary antibodies conjugated to horseradish peroxidase (HRP) at room temperature. The membrane was exposed to high performance autoradiography film (Fuji XR film; Fujifilm Corporation, Tokyo, Japan) and visualized using an enhanced chemiluminescence system (Santa Cruz Biotechnology, Inc.).

Integrated density values of the band intensities from the films were analyzed by ImageQuant 5.2 software (Molecular Dynamics, Sunnyvale, CA, USA).

### Measurement of the PMN apoptosis rate in the BALF by flow cytometry

Freshly isolated PMNs were suspended in 100 μl incubation buffer (10 mmol HEPES, 140 mmol NaCl, 5 mmol CaCl_2_ at pH 7.14), 2 μl Annexin-V-FLUOS and 2 μl propidium iodide (PI; 50 mg/l) at room temperature for 10–15 min. Subsequently, 5,000 PMNs were analyzed using a flow cytometer (Bio-Rad, Hercules, CA, USA). If positive for Annexin-V, the cells were early apoptotic cells, however, if the cells were positive for PI, the cells were necrotic. When the cells were Annexin-V- and PI-positive, the PMNs were late apoptotic necrotic cells.

### Immunohistochemistal analysis of the expression of ICAM-1 and CD11b/CD18

Mice were sacrificed and the lung lobes were dissected, fixed in 10% formaldehyde and processed for immunohistochemistry. Sections (4 μm thick) were dewaxed, rehydrated and antigen retrieval was performed with 10 mM sodium citrate (pH 6.1). The sections were then blocked with 5% bovine serum albumin (Sigma-Aldrich) for 60 min at room temperature and incubated with anti-ICAM-1 (1:200; Santa Cruz Biotechnology, Inc.) and anti-CD11b/CD18 antibodies (1:300; Santa Cruz Biotechnology, Inc.) overnight at 4°C. Subsequently, the samples were incubated with polyclonal HRP-conjugated secondary antibodies (1:100; Santa Cruz Biotechnology, Inc.) for 1 h at room temperature. The nuclei were counterstained with hematoxylin (Sigma-Aldrich) and the control slides were incubated with the same antibodies. Cover slips were mounted with 80% glycerol (ZsBio, Beijing, China).

Samples were examined using a microscope equipped with a digital camera (Hitachi, Ltd., Tokyo, Japan). ICAM-1 and CD11b/CD18 positive expression areas were quantified by densitometry using Image-Pro Plus software (Media Cybernetics, Inc., Rockville, MD, USA).

### Measurement of TNF-α, IL-1β and IL-8 expression in the BALF by ELISA

Levels of TNF-α, IL-1β and IL-8 in the BALF were determined using commercially available ELISA kits, according to the manufacturer’s instructions.

### MPO activity

The left lobe specimens of the mice were removed and preserved at −80°C. The specimens were placed in an eppendorf tube and media was added at the ratio of 1:19*,* according to the volume and weight. Samples were then centrifuged at 5,000 × g for 15 min. MPO colorimetric absorbance was measured at 460 nm, according to the manufacturer’s instructions (Wuhan Amyjet Scientific Co., Ltd., Wuhan, China), and the activity was calculated as described previously (17).

### Statistical analysis

Results are expressed as the mean ± standard deviation. Statistical analysis was performed using analysis of variance and Tukey’s test was used for comparisons among groups. P<0.05 was considered to indicate a statistically significant difference. Survival data are presented using the Kaplan-Meier method and statistical analysis was conducted with SPSS 17.0 software (SPSS, Inc., Chicago, IL, USA).

## Results

### Effect of PDTC on LPS-induced mortality in mice

**Figure 1:**
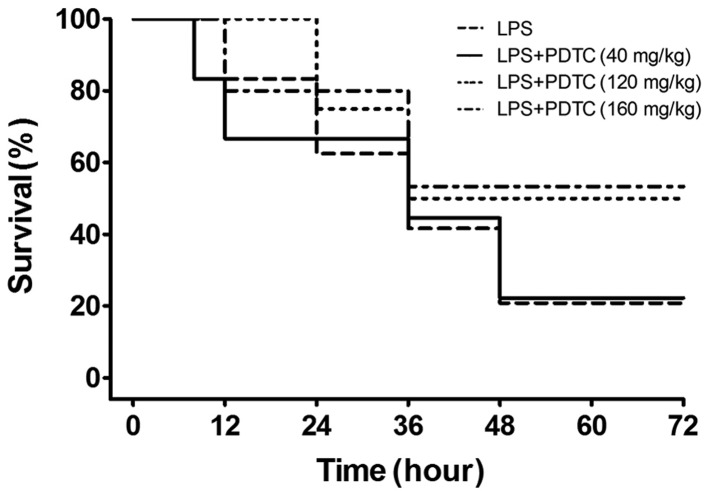
Effect of PDTC on LPS-induced mortality in mice (n=10). Mice were challenged with 20 mg/kg LPS with or without various doses of PDTC (40, 120 or 160 mg/kg, i.p.). Survival rate was observed after 12, 24, 36, 48, 60 and 72 h. The percentage survival rate of the mice was expressed using Kaplan-Meier survival curves. PDTC, pyrrolidine dithiocarbamate; LPS, lipopolysaccharide.

Shortness of breath, oral cyanosis and blood-like secretions in the nose were observed in the mice following treatment with LPS for 4 h, and the manifestations were progressive over time. The survival rate at 12, 24, 36, 48, 60 and 72 h were 100, 70, 50, 20, 20 and 20%, respectively. As shown in Fig. 1, the accumulative mortality rates during the 72 h in the 120 and 160 mg/kg PDTC treatment groups were 50 and 48%, respectively, which was significantly lower than the LPS group (80%; P<0.01). However, 40 mg/kg PDTC failed to protect against mortality (P>0.05). Therefore, 120 mg/kg PDTC was used in the further experiments.

### Pathology results of the lung tissue

**Figure 2:**
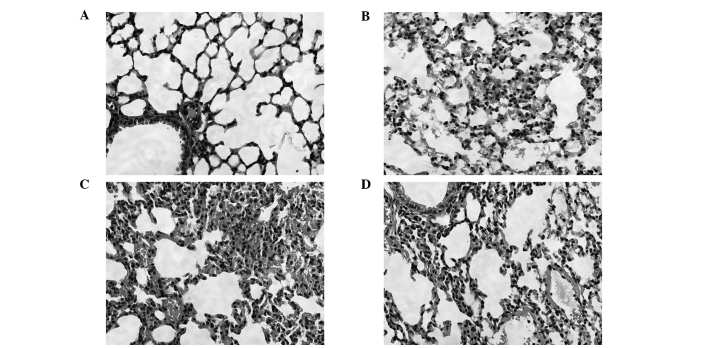
Effect of PDTC on lung histopathological changes at 4 and 24 h following saline and LPS intraperitoneal injection (HE stain; magnification, ×400). (A) Control group; (B) 4 and (C) 24 h LPS groups exhibited pulmonary edema, infiltration of inflammatory cells and alveolar damage; (D) 24 h PDTC + LPS group exhibited significantly alleviated lung injury and less damage compared with the LPS group. PDTC, pyrrolidine dithiocarbamate; LPS, lipopolysaccharide; HE, hematoxylin and eosin.

Effects of PDTC on lung histopathological changes were analyzed in the mice challenged with LPS. In the control group, the structure of the lung tissue was integrated and there were no inflammatory cells. In the 4 and 24 h LPS groups, widened lung intervals, highly congested pulmonary interstitial space, fractured alveolar walls and a large number of infiltrative inflammatory cells were observed. In addition, inflammatory cell infiltration increased and lung tissue damage became aggravated as the time progressed. In the 24 h PDTC + LPS group, there were similar symptoms to the LPS groups, however, the lung tissue injury was milder than that observed in the LPS groups (Fig. 2).

### Determination of the W/D ratio and LPI by assessing the pulmonary vascular permeability change

**Figure 3:**
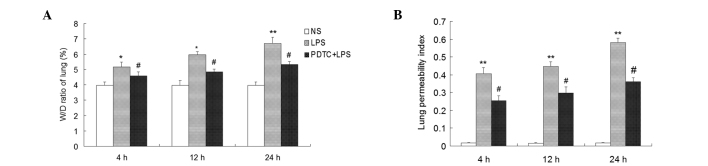
Effect of PDTC on the W/D ratio of lung tissue and protein leakage in BALF samples of LPS-induced ARDS mice. PDTC (120 mg/kg, i.p.) was administered 30 min prior to LPS administration (20 mg/kg, i.p.). Mice were anesthetized at 4, 12 and 24 h following the saline or LPS challenge. Lung tissue samples, BALF and serum were then collected immediately for (A) lung W/D ratio and (B) lung permeability index assays. Values are presented as the mean ± standard deviation. ^*^P<0.05 and ^**^P<0.01, vs. control; ^#^P<0.05, vs. LPS group (n=10). NS, normal saline; PDTC, pyrrolidine dithiocarbamate; LPS, lipopolysaccharide; W/D, wet to dry weight; BALF, bronchoalveolar lavage fluid; ARDS, acute respiratory distress syndrome.

Lung W/D ratio values significantly increased in the mice that had received an intraperitoneal injection of LPS compared with the control group at 4, 12 and 24 h (P<0.05). In addition, the W/D ratio values gradually increased as the time extended. Compared with the LPS group, the W/D ratios significantly decreased in the mice that had received an intraperitoneal injection of PDTC, but were higher compared with the control group (P<0.05; Fig. 3).

LPI values were significantly higher in the LPS group compared with the control group (P<0.01), and PDTC was shown to inhibit the LPI increase (P<0.05; Fig 3).

### Apoptosis rates and histological scores of the PMNs

**Figure 4:**
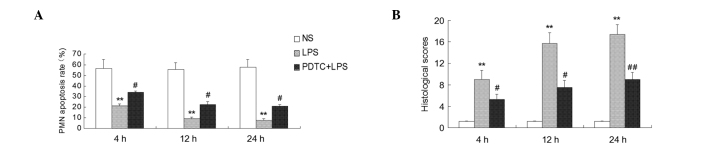
Effect of PDTC on the PMN apoptosis rate in BALF samples and the pathology scores of LPS-induced ARDS mice. Mice were anesthetized at 4, 12 and 24 h following the saline or LPS challenge. BALF and lung tissues were then collected immediately for the determination of (A) apoptosis rates and (B) histological scores. Values are presented as the mean ± standard deviation. ^*^P<0.05 and ^**^P<0.01, vs. control; ^#^P<0.05 and ^##^P<0.01, vs. LPS group (n=10). NS, normal saline; PDTC, pyrrolidine dithiocarbamate; LPS, lipopolysaccharide; BALF, bronchoalveolar lavage fluid; ARDS, acute respiratory distress syndrome; PMN, polymorphonuclear neutrophil.

As determined by flow cytometry, the PMN apoptosis rates were lower in the BALF of the LPS group compared with the control group at 4, 12 and 24 h following treatment (P<0.01). Compared with the LPS group, the PMN apoptosis rate significantly increased in the PDTC + LPS group (P<0.05; Fig. 4). Histological scores of the lung tissue were significantly increased in the mice challenged with LPS as compared with the control group (P<0.01). In addition, the histological scores were lower in the mice that had received an intraperitoneal injection of PDTC than in the LPS group at 4 and 12 h (P<0.05, P<0.01; Fig. 4), at 24 h (P<0.01; Fig. 4).

### NF-κB p65 protein expression in the cytoplasm and nuclei of lung tissue

**Figure 5:**
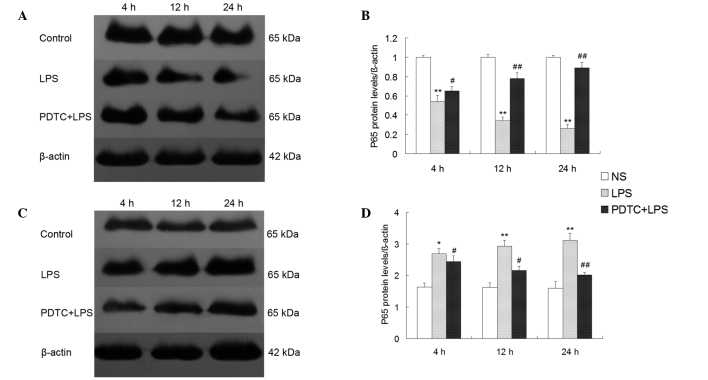
Effect of PDTC on LPS-induced NF-κB p65 expression in the cytoplasm and nucleus. Representative western blots and quantitative protein expression of NF-κB p65 in the (A and B) cytoplasm and (C and D) nucleus, respectively. Values are presented as the mean ± standard deviation. ^**^P<0.01, vs. control; ^#^P<0.05 and ^##^P<0.01, vs. LPS group (n=10). NS, normal saline; PDTC, pyrrolidine dithiocarbamate; LPS, lipopolysaccharide; NF, nuclear factor.

NF-κB p65 protein expression was significantly lower in the cytoplasm of the LPS group compared with the control group (P<0.01), however, p65 protein expression was significantly higher in the pulmonary nuclei of the LPS group compared with the control group (P<0.01). The p65 protein expression intensity in the cytoplasm of the lung tissue was increased in the PDTC + LPS group at 4 h (P<0.05; Fig. 5), at 12 and 24 h (P<0.01; Fig. 5), while p65 protein expression was decreased in the nuclei of the PDTC + LPS groupat 4 and 12 h (P<0.05; Fig. 5), at 24 h (P<0.01; Fig. 5).

### Expression of TNF-α, IL-1β and IL-8 in the BALF

**Figure 6:**
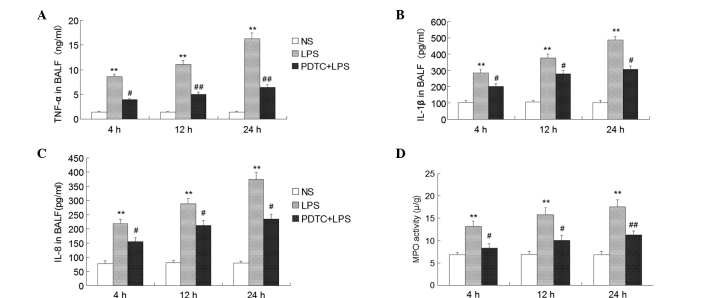
Effect of PDTC on the expression of inflammatory cytokines, including (A) TNF-α, (B) IL-1β and (C) IL-8, in the BALF and the (D) activity of MPO in the lung tissues. Values are presented as the mean ± standard deviation. ^**^P<0.01, vs. control; ^#^P<0.05 and ^##^P<0.01, vs. LPS group (n=10). NS, normal saline; PDTC, pyrrolidine dithiocarbamate; BALF, bronchoalveolar lavage fluid; MPO, myeloperoxidase; TNF, tumor necrosis factor; IL, interleukin.

The protective effect of PDTC on the overproduction of proinflammatory cytokines induced by LPS was observed. TNF-α, IL-1β and IL-8 expression levels in the BALF of the LPS group were markedly higher compared with the control group (P<0.01). However, in the group treated with 120 mg/kg PDTC prior to treatment with LPS, the expression levels of TNF-α was significantly decreased compared with the LPS group at 4 h (P<0.05; Fig. 6), at 12 and 24 h (P<0.01; Fig. 6); the expression levels of IL-1β and IL-8 were markedly decreased compared with the LPS group at 4 h (P<0.01; Fig. 6).

### Effect of PDTC on MPO activity in ARDS mice

MPO activity is an important index that can be used to evaluate the accumulation of neutrophils in lung tissue. The activity of MPO in the LPS group was markedly increased compared with the control group (P<0.01). However, this change was blocked significantly in the group treated with PDTC prior to being challenged with LPS at 4 and 12 h (P<0.05; Fig. 6), at 24 h (P<0.01; Fig. 6).

### Expression levels of CD11b/CD18 and ICAM-1

**Figure 7:**
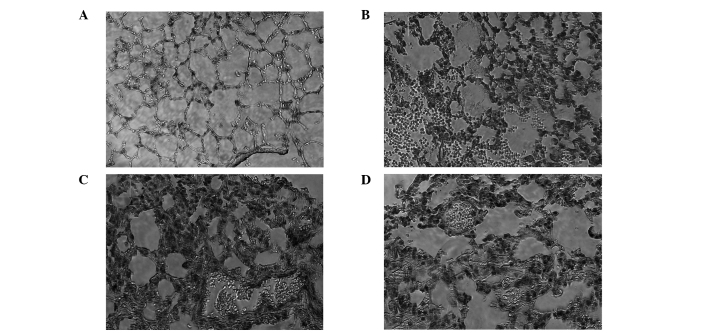
Effect of PDTC on the expression of CD11b/CD18 in the lung tissues of the (A) control, (B) 4 h LPS, (C) 24 h LPS and (D) 24 h PDTC + LPS groups. Mice were treated with PDTC (120 mg/kg, i.p.) prior to the adminstration of LPS (20 mg/kg, i.p.). Mice were anesthetized and lung tissue samples were obtained at 4 and 24 h following the saline or LPS challenge. PDTC, pyrrolidine dithiocarbamate; LPS, lipopolysaccharide.

**Figure 8:**
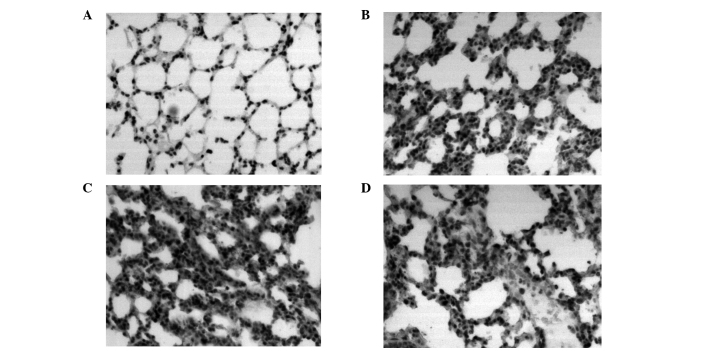
Effect of PDTC on the expression of ICAM-1 in the lung tissues of the (A) control, (B) 4 h LPS, (C) 24 h LPS and (D) 24 h PDTC + LPS groups. Mice were treated with PDTC (120 mg/kg, i.p.) prior to the administration of LPS (20 mg/kg, i.p.). Mice were anesthetized and lung tissue samples were obtained at 4 and 24 h following the saline or LPS challenge. PDTC, pyrrolidine dithiocarbamate; LPS, lipopolysaccharide; ICAM-1, intercellular adhesion molecule-1.

Immunohistochemistry results revealed that CD11b/CD18 (Fig. 7) and the ligand, ICAM-1 (Fig. 8), were highly expressed in alveolar epithelial cells and lung perivascular cells of the mild, moderate and severe LPS-induced ARDS mice. Cell morphological analysis revealed that PMNs were the predominant cells in the infiltration. However, in the control group, there was no expression in the lung tissue of the mice. The expression levels of CD11b/CD18 and ICAM-1 in the PDTC + LPS group were markedly lower compared with the LPS group.

## Discussion

Significant pathological characteristics of ARDS include the aggregation of a large number of activated neutrophils in the pulmonary vascular and mesenchyme (2). The present study systematically interpreted the PMN aggregation mechanism in the lungs. PMNs are important effector cells in an inflammatory reaction and play an important role in the occurrence and outcome of ARDS (18). Under normal conditions, circulating PMNs are not activated. PMNs exert a biological function lies in the transfer through the capillary walls into the tissue interval and the activation of PMN. IL-8 plays an important role in the migration of PMNs between the blood circulation and the blood vessels (19). CD11b/CD18, the surface adhesion molecule of PMN, is a member of the β2-integrin family and plays an important role in mediating PMN adhesion and transfer to the endothelial cells, participating in the inflammatory response (20). Under the stimulation of activated factors, including LPS, TNF-α and IL-8, CD11b/CD18 interacts with its ligand ICAM-1 on pulmonary vascular endothelial cell surfaces, activating PMNs (19) and firmly adhering to the endothelial cells. *In vitro* studies have confirmed that CD11b/CD18 is the high affinity receptor of LPS, and LPS can directly induce the expression of CD11b/CD18 (20). Furthermore, under the action of chemokines, PMNs migrate to the alveoli and interstitial space (21). Following the activation of PMNs, the cells release a large number of proinflammatory cytokines, MPO, elastase and oxygen free radicals, which results in damage to the pulmonary vascular endothelial cells and alveolar epithelial cells. In addition, an increase in pulmonary capillary membrane permeability and pulmonary interstitial edema is observed (22). The activation process is extremely crucial for PMNs to mediate lung tissue damage.

CD11b/CD18 was used as the indicator of PMN activation in the present study. There was only a small amount of CD11b/CD18 expression on the surface of the PMNs and the majority of CD11b/CD18 molecules were stored in intracellular particles at resting state (23). The expression of CD11b/CD18 on the surface of the PMNs was significantly increased in the mouse lung tissue with ARDS, and the expression was sustained at a high level (24). In addition, levels of CD11b/CD18 and ICAM-1 expression, as well as MPO activity, increased and were maintained over time, with a large number of PMNs infiltrating into the alveolar. The results indicated that expression levels of CD11b/CD18 and ICAM-1 were increased, and the interaction was the basis of PMN and endothelial cell adhesion. Thus, the interaction may play a key role in the accumulation and activation of PMNs in the lung tissue. Furthermore, this observation confirmed that the adhesion of inflammatory cells was an important mechanism in ARDS (25). IL-8 plays an important role in the process of PMN migration between the circulating blood and the extravascular (26). The present study identified that the expression of IL-8 was continuously high along with the severity of lung injury, and peaked following injection of LPS for 4 h. The results also indicated that increased IL-8 expression may promote the migration of a large number of PMNs from the peripheral blood to the lung tissue, and PMNs release toxic substances, such as MPO. In addition, IL-8 may activate PMNs and directly damage the lung tissue cells (27).

Previous studies have demonstrated that CD11b/CD18 may increase lung tissue damage by delaying inflammatory cell apoptosis and inducing the release of inflammatory cytokines from PMNs, resulting in the inflammatory cascade (14). In the current study, the apoptosis rate of PMNs was significantly reduced in the BALF of ARDS mice when lung tissue was damaged over time. In addition, the score of the damage increased with increasing pulmonary vascular permeability. The W/D ratio and LPI also increased. The results confirmed that PMN apoptosis was delayed and the survival time was prolonged following migration to the lung tissue. These changes of PMNs are one of the important mechanisms underlying ARDS inflammation, as during this time, the PMNs are active and have sustained release of inflammatory mediators and toxic contents, resulting in lung tissue damage.

As an important nuclear transcription factor, NF-κB is the intersection of multiple signaling pathways (28). A number of studies have found that the activation of NF-κB that occurs in ARDS may cause inflammatory cytokines, including adhesion molecules (CD11b/CD18, ICAM-1), chemokines (IL-8), TNF-α and IL-1β, to be expressed at the maximum level, which involves the transcriptional regulation of the activation of numerous genes (8,29). NF-κB plays an important role in the inflammatory response, oxidative stress, apoptosis and other pathological processes. TNF-α, IL-1β and IL-8, among other proinflammatory cytokines, further activate NF-κB in the lung tissue, causing the positive and negative feedback regulation of NF-κB activation to become out of balance (4). The p50/p65 heterodimer has a major physiological function during inflammation, with NF-κB p65 being the main subunit (30). The protein exists in an inactive state in the cytoplasm in the form of a dimer and directly combines with inhibitory protein IκB to form a trimeric complex. The Rel protein localization signal is exposed following stimulation with LPS, causing NF-κB to bind to specific κB sequences of DNA in the nucleus in order to regulate gene transcription and expression (9). The western blot analysis results for NF-κB p65 expression in the mouse lung tissue revealed that p65 protein expression in the cytoplasm of the lung cells was significantly decreased in the ARDS group at each time point when compared with the control group, while the p65 protein expression in the nuclei was significantly increased. These results indicated that NF-κB p65 plays an important role in the incidence and development of ARDS. In addition, NF-κB exhibits a regulatory function in PMN apoptosis in the lung tissue and inhibits the apoptosis of PMNs following activation (31).

PDTC, a specific inhibitor of NF-κB, is a dithiocarbamate of the pyrrole derivatives (32). This molecule can hinder the dissociation of the inhibitory protein IκB from the NF-κB complex via antioxidation in order to inhibit the activation of NF-κB (33). In addition, PDTC impedes the transfer of p65, an important subunit of NF-κB, to the nucleus and reduces the expression of p65 in the nucleus significantly, thus, reducing the expression levels of adhesion molecules, TNF-α, IL-1β, IL-8 and other inflammatory cytokines at the transcriptional level (34). PDTC may also directly reduce the binding ability between NF-κB and DNA, and obstruct the signaling pathway activated by NF-κB, thus, decreasing the production of CD11b/CD18, ICAM-1 and chemokines (35). The present study found that application of PDTC suppressed the activation and transfer of NF-κB p65 in the lung tissue and increased the expression of cytoplasmic p65 protein, while decreasing the expression in the nucleus. In addition, the expression levels of CD11b/CD18, ICAM-1, TNF-α, IL-1β and IL-8 were significantly reduced in the lung tissue cells, while the apoptosis rate of the PMNs was significantly increased and the activity of MPO was less compared with the LPS group. In combination with the determination of lung tissue pathology and pulmonary permeability results, PDTC may reduce the expression of adhesion molecules and chemokines by inhibiting the activation of NF-κB and the activation of PMNs and causing the release of MPO. PDTC may also reduce the pulmonary capillary permeability and the infiltration of inflammatory cells. Therefore, the NF-κB signaling pathway is hypothesized to be an important intervention in targeting the regulation of PMN aggregation, and controlling the activation of NF-κB may become a key strategy for the treatment of ARDS.

In conclusion, the present study demonstrated that the transfer and activation of NF-κB from the cytoplasm to the nucleus in the lung tissue was induced by LPS, which then initiated the synthesis and release of cell adhesion molecules and chemokines, causing the accumulation of a large number of PMNs in the lung tissue. However, pretreatment with PDTC partially reduced the lethality in LPS-induced mice by attenuating the lung tissue edema and damage, the production of inflammatory cytokines, the neutrophil influx to the lung and the overactivation of NF-κB, promoting PMN apoptosis via the NF-κB pathway. These results indicate that the NF-κB signaling pathway may be an important intervention in targeting the regulation of PMN aggregation in the lungs.

## References

[1] Rubenfeld GD, Caldwell E, Peabody E (2005). Incidence and outcomes of acute lung injury. N Engl J Med.

[2] Matthay MA, Zimmerman GA (2005). Acute lung injury and the acute respiratory distress syndrome: four decades of inquiry into pathogenesis and rational management. Am J Respir Cell Mol Biol.

[3] Kuo MY, Liao MF, Chen FL (2011). Luteolin attenuates the pulmonary inflammatory response involves abilities of antioxidation and inhibition of MAPK and NF-κB pathways in mice with endotoxin-induced acute lung injury. Food Chem Toxicol.

[4] Bhatia M, Moochhala S (2004). Role of inflammatory mediators in the pathophysiology of acute respiratory distress syndrome. J Pathol.

[5] Giebelen IA, van Westerloo DJ, LaRosa GJ (2007). Local stimulation of alpha7 cholinergic receptors inhibits LPS-induced TNF-alpha release in the mouse lung. Shock.

[6] Lynn WA, Raetz CR, Qureshi N, Golenbock DT (1991). Lipopolysaccharide-induced stimulation of CD11b/CD18 expression on neutrophils. Evidence of specific receptor-based response and inhibition by lipid A-based antagonists. J Immunol.

[7] Smith CW, Marlin SD, Rothlein R, Toman C, Anderson DC (1989). Cooperative interactions of LFA-1 and Mac-1 with intercellular adhesion molecule-1 in facilitating adherence and transendothelial migration of human neutrophils in vitro. J Clin Invest.

[8] Everhart MB, Han W, Sherrill TP (2006). Duration and intensity of NF-kappaB activity determine the severity of endotoxin-induced acute lung injury. J Immunol.

[9] Nathens AB, Bitar R, Davreux C, Bujard M (1997). Pyrrolidine dithiocarbamate attenuates endotoxin-induced acute lung injury. Am J Respir Cell Mol Biol.

[10] Lindström P, Lerner R, Palmblad J, Patarroyo M (1990). Rapid adhesive responses of endothelial cells and of neutrophils induced by leukotriene B4 are mediated by leucocytic adhesion protein CD18. Scand J Immunol.

[11] Weber C, Erl W, Pietsch A, Weber PC (1995). Aspirin inhibits nuclear factor-kappa B mobilization and monocyte adhesion in stimulated human endothelial cells. Circulation.

[12] Borregaard N, Sørensen OE, Theilgaard-Mönch K (2007). Neutrophil granules: a library of innate immunity proteins. Trends Immunol.

[13] Leindler L, Morschl E, László F (2004). Importance of cytokines, nitric oxide, and apoptosis in the pathological process of necrotizing pancreatitis in rats. Pancreas.

[14] Harkin DW, Marron CD, Rother RP, Romaschin A (2005). C5 complement inhibition attenuates shock and acute lung injury in an experimental model of ruptured abdominal aortic aneurysm. Br J Surg.

[15] Hashimoto N, Kawabe T, Imaizumi K (2004). CD40 plays a crucial role in lipopolysacharide-induced acute lung injury. Am J Respir Cell Mol Biol.

[16] Haddad JJ, Safieh-Garabedian B, Saadé NE, Lauterbach R (2002). Inhibition of glutathione-related enzymes augments LPS-mediated cytokine biosynthesis; involvement of an IkappaB/NF-kappaB-sensitive pathway in the alveolar epithelium. Int Immunopharmacol.

[17] Fan J, Marshall JC, Jimenez M (1998). Hemorrhagic shock primes for increased expression of cytokine-induced neutrophil chemoattractant in the lung: role in pulmonary inflammation following lipopolysaccharide. J Immunol.

[18] Grommes J, Soehnlein O (2011). Contribution of neutrophils to acute lung injury. Mol Med.

[19] Woodfin A, Voisin MB, Nourshargh S (2010). Recent developments and complexities in neutrophil transmigration. Curr Opin Hematol.

[20] Triantafilou M, Triantafilou K (2002). Lipopolysaccharide recognition: CD14, TLRs and the LPS-activation cluster. Trends Immunol.

[21] Meliton AY, Muñoz NM, Meliton LN, Binder DC (2010). Cytosolic group IVa phospholipase A2 mediates IL-8/CXCL8-induced transmigration of human polymorphonuclear leukocytes in vitro. J Inflamm (Lond).

[22] Wang X, Wang Y, Zhao X, Andersson R (2009). Potential effects of peroxisome proliferator-activated receptor activator on LPS-induced lung injury in rats. Pulm Pharmacol Ther.

[23] Hoshino H, Laan M, Sjöstrand M (2000). Increased elastase and myeloperoxidase activity associated with neutrophil recruitment by IL-17 in airways in vivo. J Allergy Clin Immunol.

[24] Arnaout MA (1990). Structure and function of the leukocyte adhesion molecules CD11/CD18. Blood.

[25] Bhatia RK, Pallister I, Dent C (2005). Enhanced neutrophil migratory activity following major blunt trauma. Injury.

[26] Pallister I, Dent C, Topley N (2002). Increased neutrophil migratory activity after major trauma: a factor in the etiology of acute respiratory distress syndrome. Crit Care Med.

[27] Nooteboom A, van der Linden CJ, Hendriks T (2004). Modulation of adhesion molecule expression on endothelial cells after induction by lipopolysaccharide-stimulated whole blood. Scand J Immunol.

[28] Chen X, Yang X, Liu T (2012). Kaempferol regulates MAPKs and NF-κB signaling pathways to attenuate LPS-induced acute lung injury in mice. Int Immunopharmacol.

[29] Tanaka S, Nishiumi S, Nishida M, Mizushina Y (2010). Vitamin K3 attenuates lipopolysaccharide-induced acute lung injury through inhibition of nuclear factor-κB activation. Clin Exp Immunol.

[30] Ross SD, Kron IL, Gangemi JJ (2000). Attenuation of lung reperfusion injury after transplantation using an inhibitor of nuclear factor-κB. Am J Physiol Lung Cell Mol Physiol.

[31] Kupfner JG, Arcaroli JJ, Yum HK (2001). Role of NF-kappaB in endotoxemia-induced alterations of lung neutrophil apoptosis. J Immunol.

[32] Cuzzocrea S, Chatterjee PK, Mazzon E (2002). Pyrrolidine dithiocarbamate attenuates the development of acute and chronic inflammation. Br J Pharmacol.

[33] Liu SF, Malik AB (2006). NF-kappa B activation as a pathologic mechanism of septic shock and inflammation. Am J Physiol Lung Cell Mol Physiol.

[34] Németh ZH, Haskó G, Vizi ES (1998). Pyrrolidine dithiocarbamate augments IL-10, inhibits TNF-alpha, MIP-1alpha, IL-12, and nitric oxide production and protects from the lethal effect of endotoxin. Shock.

[35] Roy A, Jana A, Yatish K (2008). Reactive oxygen species up-regulate CD11b in microglia via nitric oxide: Implications for neurodegenerative diseases. Free Radic Biol Med.

